# Gene Expression Patterns Distinguish Mortality Risk in Patients with Postsurgical Shock

**DOI:** 10.3390/jcm9051276

**Published:** 2020-04-28

**Authors:** Pedro Martínez-Paz, Marta Aragón-Camino, Esther Gómez-Sánchez, Mario Lorenzo-López, Estefanía Gómez-Pesquera, Rocío López-Herrero, Belén Sánchez-Quirós, Olga de la Varga, Álvaro Tamayo-Velasco, Christian Ortega-Loubon, Emilio García-Morán, Hugo Gonzalo-Benito, María Heredia-Rodríguez, Eduardo Tamayo

**Affiliations:** 1 Department of Surgery, Faculty of Medicine, University of Valladolid, 47005 Valladolid, Spain; pedrojose.martinez@uva.es (P.M.-P.); esthergzam@hotmail.com (E.G.-S.); mariolorenzo17@yahoo.es (M.L.-L.); egp29@hotmail.com (E.G.-P.); maria_her_05@hotmail.com (M.H.-R.); eduardo.tamayo@uva.es (E.T.); 2 BioCritic. Group for Biomedical Research in Critical Care Medicine, 47005 Valladolid, Spain; mrt.aragon@gmail.com (M.A.-C.); rociolopezherrero@hotmail.com (R.L.-H.); belensq93@gmail.com (B.S.-Q.); olga.v.m91@gmail.com (O.d.l.V.); alvarotv1993@gmail.com (A.T.-V.); christlord26@gmail.com (C.O.-L.); hgonzalob@saludcastillayleon.es (H.G.-B.); 3 Anesthesiology and Resuscitation Service, University Clinical Hospital, 47003 Valladolid, Spain; 4 Haematology and Hemotherapy Service, University Clinical Hospital, 47003 Valladolid, Spain; 5 Cardiac Surgery Service, University Clinical Hospital, 37007 Salamanca, Spain; 6 Cardiology Service, University Clinical Hospital, 47003 Valladolid, Spain; 7 Institute of Health Sciences of Castile and Leon (IECSCYL), 47003 Valladolid, Spain; 8 Anesthesiology and Resuscitation Service, University Hospital, 37007 Salamanca, Spain

**Keywords:** transcriptomic profile, mortality, postsurgical shock, sepsis, microarray, biomarker

## Abstract

Nowadays, mortality rates in intensive care units are the highest of all hospital units. However, there is not a reliable prognostic system to predict the likelihood of death in patients with postsurgical shock. Thus, the aim of the present work is to obtain a gene expression signature to distinguish the low and high risk of death in postsurgical shock patients. In this sense, mRNA levels were evaluated by microarray on a discovery cohort to select the most differentially expressed genes between surviving and non-surviving groups 30 days after the operation. Selected genes were evaluated by quantitative real-time polymerase chain reaction (qPCR) in a validation cohort to validate the reliability of data. A receiver-operating characteristic analysis with the area under the curve was performed to quantify the sensitivity and specificity for gene expression levels, which were compared with predictions by established risk scales, such as acute physiology and chronic health evaluation (APACHE) and sequential organ failure assessment (SOFA). *IL1R2*, *CD177*, *RETN*, and *OLFM4* genes were upregulated in the non-surviving group of the discovery cohort, and their predictive power was confirmed in the validation cohort. This work offers new biomarkers based on transcriptional patterns to classify the postsurgical shock patients according to low and high risk of death. The results present more accuracy than other mortality risk scores.

## 1. Introduction

Shock is defined as acute circulatory failure with inadequate or inappropriate tissue perfusion, resulting in generalized cellular hypoxia [1]. This condition is a common complication of critical illness in patients in intensive care units (ICUs), who are undergoing major surgery. The rate of postsurgical ICU admissions has increased each year, with a proportional increase in the severity of co-existing disease, ICU-specific interventions, and the number of ICU beds [2,3,4]. Despite a decrease in the mortality rate in ICUs in recent years, its rate is still the highest of all the hospital units [5,6]. In the postsurgical ICU, the most common cause of death is shock, including septic shock and hypovolemic shock, with multiple organ failure [7], reaching 30–50% of mortality in severe sepsis and 50–60% in septic shock [8,9]. Compounding this situation, the average daily cost of an ICU bed is threefold higher than one on a general ward [10].

There are different ICU scoring systems for predicting the likelihood of mortality, such as the acute physiology and chronic health evaluation (APACHE), sequential organ failure assessment (SOFA), and simplified acute physiology score (SAPS). Nevertheless, despite the demonstration of good discrimination by these score systems, they are used in only 10–15% of US ICUs because they also depend on the reliability and predictions of physicians [11]. These risk scores are based on the use of physiological and other clinical data at the organ level, and yet, they do not take into account molecular changes that may occur at the cellular level. In this sense, the application of gene expression profiles to evaluate patient survival has been developed, mainly for cancer patients [12], with the following criteria for gene selection: association with outcomes, accuracy, and reproducibility in an independent cohort and the independency of its prognostic value from other standard factors in multivariate analysis [13]. However, less is known about the analysis of transcript patterns as a complement to clinical management in ICU patients. In the last years, some studies involving septic patients correlated gene expression signatures to organ failure and mortality [14,15,16,17], but any previous work has analyzed the mortality likelihood only in postsurgical shock patients. Therefore, the development of a molecular test based on gene expression patterns, following the aforementioned criteria, could provide a prognostic tool that improves risk stratification and mortality prediction in patients with postsurgical shock. Based on these considerations, this study determined whether gene expression signatures could predict mortality in patients with postsurgical shock and whether reliable biomarkers could be identified.

## 2. Experimental Section

### 2.1. Patient Selection and Clinical Data

The current study was performed in the postsurgical ICU of Hospital Clínico Universitario de Valladolid, Spain. It involved two cohorts of adult patients after major surgery between January 2014 and December 2018. Gene expression profiles by microarray analysis were obtained in one of the cohorts, the discovery cohort. The other cohort, the validation cohort, was used to validate the results obtained from the discovery cohort by quantitative real-time polymerase chain reaction (qPCR). For both cohorts, all patients had a lactate value >2 mM with persisting hypotension requiring vasopressors to maintain MAP ≥65 mmHg anytime in the first 24 postoperative hours, and the main outcome was measured as survival or non-survival 30 days after the operation. On the other hand, non-Caucasians, pregnant women, patients in agonizing state, and those in a state of limitation of the therapeutic efforts were excluded from the final analysis. This study followed the code of ethics of the World Medical Association (Declaration of Helsinki). It was also approved by the Scientific Committee for Clinical Research of Hospital Clínico Universitario de Valladolid, and patients or legal representatives provided informed written consent before recruitment. A survey was used to collect clinical data, including medical history; physical examinations; and hematological, biochemical, radiological, and microbiological investigations.

### 2.2. Sample Collection and RNA Extraction

Whole blood samples were collected from patients using PAXgene venous blood vacuum collection tubes (Becton Dickinson, Franklin Lakes, NJ, USA) within 24 h of ICU admission. Total RNA was extracted and purified from blood samples using the PAXgene Blood RNA System (PreAnalytix, Hombrechtikon, Switzerland) and a RNeasy Mini Kit (Qiagen, Hilden, Germany), following the manufacturers’ protocols. The quality of the total RNA was assessed with an RNA Experion Bioanalyser (Bio-Rad, Hercules, CA, USA), and the quantity was evaluated by absorbance on a NanoDrop 1000 Spectrophotometer (NanoDrop Technologies, Wilmington, DE, USA). Up to 1.75 µg of each RNA sample was concentrated with an RNeasy MinElute Cleanup Kit (Qiagen, Hilden, Germany) and eluted in a final volume of 10 μL, according to the manufacturer’s instructions. Purified RNAs were stored at −80 °C.

### 2.3. Microarray Processing and Data Analysis

Cyanine 3-CTP-labelled cRNA was generated from 100 ng total RNA using a Quick Amp Labelling Kit (Agilent, Palo Alto, CA, USA), according to the manufacturer’s instructions. Following the One-Color Microarray-Based Gene Expression Analysis Protocol version 5.7 (Agilent, Santa Clara, CA, USA), 3 µg labelled cRNA was hybridized to a Whole Human Genome Oligo Microarray (GPL10487; Agilent, Palo Alto, CA, USA), which contained 41,000 unique human genes and transcripts. Arrays were scanned in an Agilent G2565BA Microarray Scanner System (Agilent, Wilmington, DE, USA), according to the manufacturer’s protocol, and data were extracted with Agilent Feature Extraction Software version 9.5.3, using Agilent protocol GE1-v5_95_Feb07 and the QC Metric Set GE1_QCMT_Jan08. Raw data files were imported into an R-Bioconductor programming environment using the read.maimages function from the limma package. For repeat probes, median values were used. Preprocessing involved background correction using the normexp (‘saddle’) method, with an offset value of 50. Normalization between the arrays was performed by the quantile method. The expression matrix was summarized for further analysis by the selection of the top decile of probes in terms of variance. A clustering procedure was performed on the expression matrix to define relevant patient clusters (column-wise clustering) and gene clusters (row-wise clustering). The clustering function was hclust from the R stats package, using Euclidean distance and the Ward.D2 aggregation method. Patient clusters were compared in terms of survival analysis. We assessed differential expression on the expression matrix analysis using the lmFit function from the limma package in order to obtain log-fold changes of the genes that could best distinguish between clusters of patients, assuming that the clustering performed according to the class discovery method. The most significant gene clusters were functionally validated by input into the Search Tool for the Retrieval of Interacting Genes website version 11 (STRING; Swiss Institute of Bioinformatics) to test for significant protein–protein interactions (PPIs) and for enrichment of gene ontology (GO) tags. For STRING analysis, the high interaction confidence score of 0.7 was set as a threshold value.

Most differentially expressed genes (DEGs) between low- and high-risk patients were tested for their ability to predict mortality in the validation cohort, as described below. The microarray dataset was deposited in the National Center for Biotechnology Information (NCBI) Gene Expression Omnibus (GEO), accessible through GEO Series accession number GSE132897.

### 2.4. Quantitative Real-Time Polymerase Chain Reaction (qPCR)

To assess the reliability of data obtained from the microarray analysis, genes *OLFM4*, *CD177*, *RETN*, and *IL1R2* were selected based on their fold changes in expression and *p*-values. The expression levels for these genes were evaluated by qPCR in the validation cohort. cDNA was obtained by reverse transcription using an iScript Advanced cDNA Synthesis Kit (Bio-Rad, Hercules, CA, USA) and RNA isolated from the patients. The cDNA was used as a template for qPCR to evaluate the mRNA expression profiles of the aforementioned genes in patients of the survival group and those who died within 30 days of their operation. qPCR was performed in a CFX96 thermocycler (Bio-Rad, Hercules, CA, USA) using PrimeTime Gene Expression Master Mix and cycling conditions of an initial denaturation at 95 °C for 3 min, 45 cycles of denaturation at 95 °C for 15 s, and annealing and elongation at 62 °C for 15 s. In each case, the gene expression patterns of surviving patients were compared with those observed in patients who died within 30 days of their operation, after normalization with the actin gene, which was employed as a constitutively expressed reference gene. The sequences of primers for the selected genes are listed in Table 1.

The PCR amplification efficiency was established using calibration curves. For each gene, a standard curve based on five dilutions from an equimolar mix of cDNA samples was produced in triplicate. Each sample was run in triplicate wells. The cycle threshold (Ct) values were obtained with Bio-Rad CFX Maestro software (Bio-Rad, Hercules, CA, USA) and converted to relative gene expression levels using the 2^−ΔΔCt^ method.

### 2.5. Statistical Analysis

All the statistical analyses were performed using SPSS Statistics for Windows version 24.0 (IBM, Armonk, NY, USA) and R statistical package version 3.6.0 (The R Foundation, Vienna, Austria). Categorical variables were evaluated using Pearson’s χ^2^ test, and continuous variables were analyzed by Student’s t-test to find qualitative statistical significance. Normal distribution and variance homogeneity of data were assessed using the Kolmogorov–Smirnov and Levene’s tests, respectively. The Kaplan–Meier method with the log-rank test was used for survival analyses. Receiver operating characteristic (ROC) analysis with area under the curve (AUC) was calculated to quantify the sensitivity and specificity of gene expression levels. A forward multivariate logistic process was used to add the best-performing clinical parameters to our model. The optimal cut-off value with higher mortality was obtained using classification and regression tree (CART) Analysis, which is ideally suited to the generation of clinical decision making [18]. The ability of this cut-off value to predict 30-day mortality was further evaluated by using multivariate logistic regression analysis. Model calibration was assessed using the Hosmer–Lemeshow test. In all cases, a *p*-value ≤ 0.05 was considered to indicate statistical significance.

## 3. Results

### 3.1. Patient Characteristics

The clinical characteristics of postsurgical patients enrolled in this study are described in Table 2. Surviving patients and non-surviving patients 30 days after surgery in the discovery and validation cohorts were not significantly different for most variables; however, they were significantly different in terms of the length of hospital stay and lactate levels.

### 3.2. Identification of Biomarker Genes for Mortality Risk after Surgery

**Figure 1:**
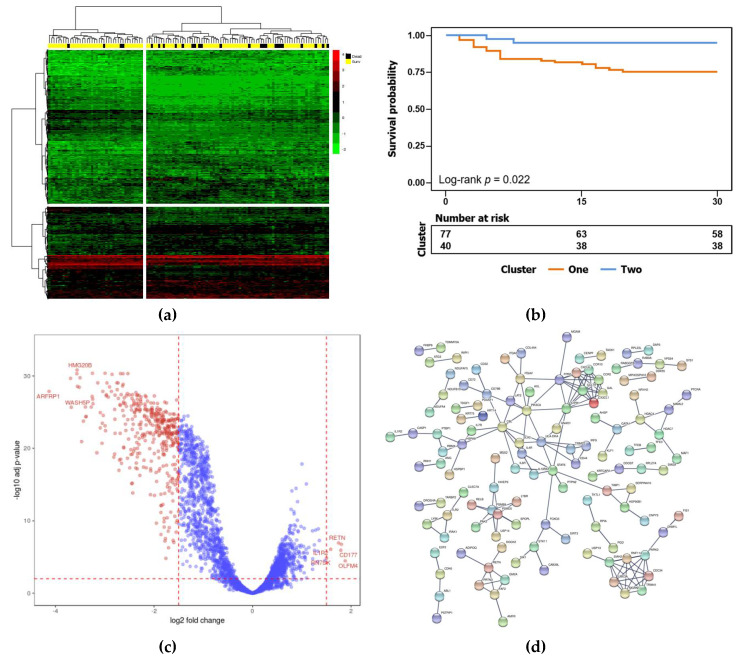
Identification of biomarker genes from gene expression data. (**a**) Heat map plot of genes of interest. Rows represent the gene expression value and columns represent the samples. The scale bar represents the intensity of expression of transcripts, with red indicating overexpressed transcripts and green representing underexpressed transcripts. The top bar indicates surviving (yellow) and non-surviving patients (black); (**b**) Kaplan–Meier plot showing survival probability of two groups of patients clustered by risk mortality. The numbers below the graph indicate the number of patients at risk of death in each group; (**c**) volcano plot of the differentially expressed genes, with red coloring for fold changes >1.5 and *p*-value < 0.01; (**d**) protein–protein interaction network of differentially expressed genes.

A graphical representation of the expression matrix of the top decile variant genes is shown in Figure 1a, after row-wise (genes) and column-wise (patients) clustering. The matrix is divided in two blocks in columns and rows, since this division provided the highest step in distance between clusters, showing that dividing patients and genes in two clusters was the optimal grouping. The color scale suggests differences in RNA levels of genes between the two clusters of patients. The color annotation bar (yellow and black) shows the distribution of survivors and non survivors in each cluster of patients. The statistical significance of the difference in survival between the two patient clusters was demonstrated by Kaplan-Meier survival analysis (Figure 1b). The Kaplan-Meier plot shows significant differences in the prognosis of patients in Cluster 1 (high risk) and Cluster 2 (low risk), suggesting that the expression pattern could distinguish between a high and low risk of death. The volcano plot shows all the genes in the expression matrix, ranking them as upregulated (right end) or downregulated (left end) in the patients of the cluster at high risk of mortality. The log-fold changes and their *p*-values were determined, as well as the association with the row-wise clustering of the expression matrix (Table S1). Cluster 1 was the smaller-sized cluster, the functional validation of which was performed on STRING. The PPI network showed a total of 301 nodes and 42 edges, with a PPI score of >0.4 based on the STRING database (Figure 1d).

### 3.3. Validation of Biomarker Genes in the Validation Cohort

**Figure 2:**
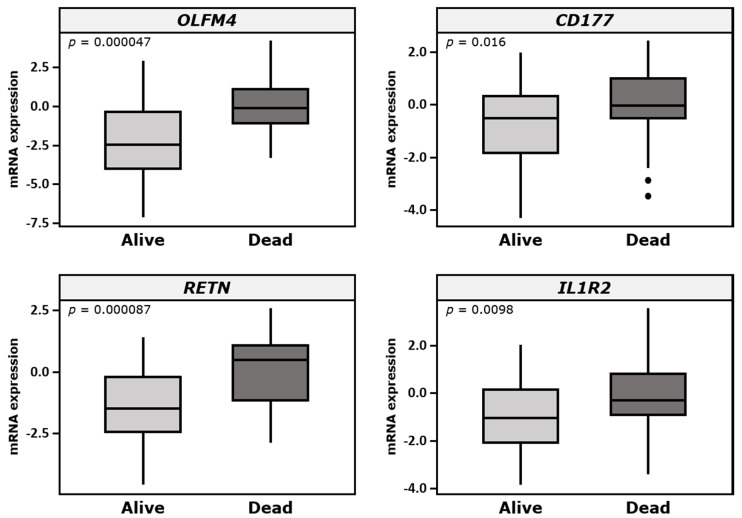
Relative mRNA levels of *OLFM4*, *CD177*, *RETN*, and *IL1R2* in surviving patients and non-surviving patients as measured by qPCR. The primers and reference genes are given in the Methods section. Horizontal lines within the boxes represent the median, and the boundaries of the boxes indicate the 25th and 75th percentiles, while the whiskers indicate the highest and lowest values. The Y-axis represents the RNA expression levels in arbitrary units and logarithmic scale. qPCR, quantitative real-time polymerase chain reaction.

*OLFM4*, *CD177*, *RETN*, and *IL1R2* were selected from the microarray analysis for validation by qPCR in an independent study cohort (validation cohort) to evaluate the robustness of these genes as candidate biomarkers. qPCR data showed similar expression patterns for these genes, with a significant upregulation in non-surviving patients compared with surviving patients, confirming the results obtained from microarray analysis (Figure 2).

### 3.4. Mortality Prediction by Biomarkers Compared to Classical Risk Scales

**Figure 3:**
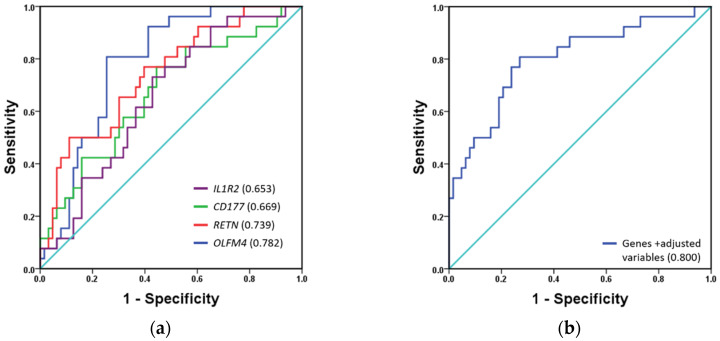
Quantification of mortality prediction accuracy by ROC AUC. (**a**) ROC AUC analysis of gene expression; (**b**) ROC AUC analysis of multivariate regression model that includes gene expression, emergency, sex, and age data, as well as creatinine, bilirubin, lactate, and white blood cell levels, as adjusted variables. ROC, receiver operating characteristic; AUC, area under the curve.

We assessed the ability of gene expression levels to predict the likelihood of mortality by comparing our results with the most used mortality risk scores, such as APACHE and SOFA. The clinical parameters to calculate the APACHE and SOFA values were taken at the same time blood was collected for gene expression assays. Initially, selected genes were evaluated by ROC curve analysis of the validation cohort, with AUC used to quantify its accuracy (Figure 3a). *OLFM4* showed the highest AUC (0.782; 0.687–0.877), followed by *RETN* (0.739; 0.628–0.850), *CD177* (0.669; 0.544–0.794), and *IL1R2* (0.653; 0.535–0.771). Multivariate regression modeling with these genes improved the AUC value to 0.760 (0.649–0.872; Figure 3b). These results were better than AUC values for APACHE and SOFA, and other classical biomarkers, such as lactate, procalcitonin, and C-reactive protein (Table 3). Notably, logistic regression modeling was performed, including the gene cluster, emergency, sex, and age data, as well as creatinine, bilirubin, lactate, and white-blood-cell levels, with the aim of improving the AUC value. This model had a very good accuracy for patients in this current study, increasing the AUC value to 0.800 (0.693–0.906; Figure 3b).

**Figure 4:**
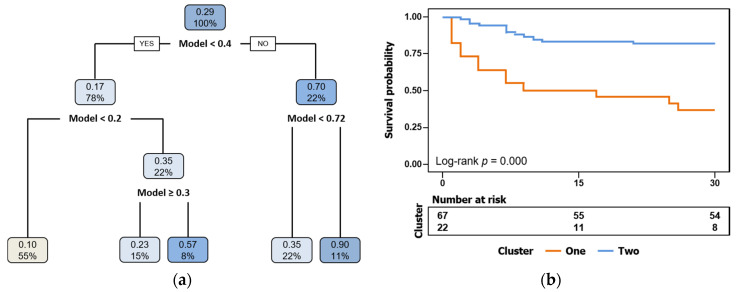
Survival analysis based on regression model. (**a**) Risk mortality tree generated by classification and regression tree (CART) analysis; (**b**) Kaplan–Meier curve for overall survival based on CART analysis.

For survival analysis, CART was employed to determine the optimal cut-off value with higher risk of mortality in patients with postsurgical shock (Figure 4a), and subsequently, a Kaplan–Meier plot was performed (Figure 4b), revealing significant differences among groups by the log-rank test.

## 4. Discussion

This study analyzed gene expression patterns in patients who developed shock after a major surgery, comparing those that survived for 30 days post operation and those that died. It showed: (i) transcriptomic profiling could predict mortality in postoperative patients with shock; (ii) four genes were identified as single efficient biomarkers that distinguished between low and high risk of death; and (iii) the gene expression cluster can predict mortality better than classical mortality risk scores.

This current study succeeded in finding a differential gene expression pattern that predicted mortality in postoperative patients with shock. The most significant cluster of genes could be arranged as a significant PPI network. This suggested a specific gene expression signature was associated with the differentiation between high and low risk clusters of patients. The most significantly enriched GO tag was immune system process (GO term 0002376). Besides this gene pattern, the study aimed to discover single efficient biomarkers. The estimation of log-fold changes between low- and high-risk patients for every gene, regardless of gene clustering, showed a number of overexpressed genes, with the top four being DEGs *OLFM4*, *CD177*, *RETN*, and *IL1R2*. We gave higher importance to these overexpressed genes on account of their functional profile, according to reactome pathways. These pathways focus on immune responses, fibrin clot formation, and responses to metabolic stress. By contrast, functional profiles of the many more underexpressed genes were far less indicative, with nearly half missing functional tagging. Keratinization was associated with a few underexpressed genes, though these were unlikely to be related to the well-established physiopathology of shock, in sharp contrast with the overexpressed genes. For this reason, the overexpressed genes rather than the abundant and highly significantly underexpressed genes were used in the PCR-based validation phase on the independent cohort (Figure 1c).

Previous work has reported *OLFM4* gene expression as a biomarker for sepsis diagnosis [19]; however, this work compared postsurgical septic patients versus postsurgical control patients, who did not show any signs or symptoms. Hence, the main strength of this study is the comparison between septic shock and non-septic shock postoperative patients, who showed higher SOFA score than Almansa et al. report [19] for hyperlactatemia.

The selected gene cluster could predict mortality in the independent validation cohort. Thus, the AUC values for each of the selected genes were better than the AUC values of other classical mortality risk scores, such as APACHE and SOFA. Previous reports suggested that gene expression patterns could be used as biomarkers to predict the survival of patients with different illnesses, such as leukemia, gastric cancer, hepatitis, and biliary atresia, and this current study added postoperative shock to this list [20,21,22,23]. An advantage of this current study was that it identified a small number of genes, which would make the procedures easily transferable to hospital-based clinical laboratories, where PCR is a fast, cheap, accurate, and reliable technique used on a daily basis. Moreover, in order to better stratify the mortality risk on postsurgical shock patients, CART analysis was used to perform a decision tool to classify patients, and a Kaplan–Meier plot based on CART results was created to confirm the ability of the gene cluster to predict mortality.

In addition, survival analysis based on gene expression can also help to understand the outcome in terms of the underlying biology [24]. In this regard, the top four overexpressed genes identified in the current study were related to the immune system. This might suggest that patients with shock who experienced a higher mortality were actually suffering the early stages of an unrecognized infection, which might even cause death before it could be diagnosed [25].

Finally, three important limitations of this study should also be acknowledged. First, this study did not analyze the evolution of gene expression patterns over time. Second, it is a single-center study; therefore, it did not assess possible inter-hospital variation, indicating the value of extending it to a multicenter study. Third, because of the nature of the samples, the gene expression analyses of white blood cells mainly provided insight into immune pathways regulated at the mRNA level.

## 5. Conclusions

Transcript profiling can predict the survival of patients with postsurgical shock. This study provided a transcript-based tool to classify patients as “low risk” or “high risk” with regard to survival. However, further research is needed to validate these findings in independent prospective cohorts and establish the clinical application of this prognostic system.

## Figures and Tables

**Table 1:** Primers used for qPCR of genes from human.

Gene	Forward (5′-3′)	Reverse (5′-3′)	Efficiency
*Actin*	CCTTGCACATGCCGGAG	ACAGAGCCTCGCCTTTG	87.2%
*IL1R2*	GCATCTGTATTCTCAAAAACTCTGA	GGTGCTCTGTGGCTTCTG	96.9%
*CD177*	AAGAGATTACCAGCCACAGAC	GCTGAACTGTCCCAAACTG	90.0%
*RETN*	GCCGGATTTGGTTAGCTGA	CATGGAGCACAGGGTCTTG	99.7%
*OLFM4*	TGCTGATGTTCACCACACC	CTGAAGACCAAGCTGAAAGAGT	92.2%

qPCR: quantitative real-time polymerase chain reaction.

**Table 2:** Characteristics of postsurgical patients.

	Discovery Cohort		Validation Cohort			
	Surviving (*n* = 88)	Non-Surviving (*n* = 29)	*p*	Surviving (*n* = 79)	Non-Surviving (*n* = 33)	*p*
**Characteristics**						
Age	69.15	71.86	0.297	69.06	72.70	0.108
Male (*n* (%))	55 (63)	18 (62)	0.967	50 (63)	23 (70)	0.517
**Comorbidities (*n* (%))**						
High blood pressure	64 (73)	19 (66)	0.458	46 (58)	23 (70)	0.255
Chronic cardiovascular disease	53 (60)	14 (48)	0.259	20 (25)	10 (30)	0.587
Chronic respiratory disease	14 (16)	5 (17)	0.866	14 (18)	8 (24)	0.428
Chronic renal failure	10 (11)	6 (21)	0.205	5 (6)	3 (9)	0.605
Chronic hepatic failure	3 (3)	0 (0)	0.314	1 (1)	0 (0)	0.516
Diabetes mellitus	25 (28)	7 (24)	0.655	16 (20)	6 (18)	0.801
Cancer	23 (26)	5 (17)	0.330	17 (22)	9 (27)	0.511
Immunosuppression	4 (5)	1 (3)	0.800	4 (5)	0 (0)	0.188
**Time course and outcome**						
Length of hospital stay	30.51	18.31	0.011	37.22	12.21	0.000
Length of ICU stay	8.26	7.03	0.525	10.58	6.61	0.021
Mortality (% (7 days))	0 (0)	14 (48)	0.000	0 (0)	15 (45)	0.000
Mortality (% (15 days))	0 (0)	21 (72)	0.000	0 (0)	28 (85)	0.000
**Type of surgery (*n* (%))**						
Cardiac surgery	54 (61)	14 (48)	0.215	34 (43)	15 (45)	0.814
General surgery	26 (30)	12 (41)	0.238	35 (44)	15 (45)	0.911
Others	8 (9)	3 (11)	1.000	10 (13)	3 (10)	0.755
**Source of infection (*n* (%))**						
Respiratory tract	19 (22)	9 (31)	0.301	20 (25)	8 (24)	0.905
Abdomen	15 (17)	5 (17)	0.981	17 (22)	8 (24)	0.752
Urinary tract	12 (14)	4 (14)	0.983	13 (16)	2 (6)	0.141
Surgical site	22 (25)	5 (17)	0.390	21 (27)	7 (21)	0.550
Bacteremia	23 (26)	7 (24)	0.831	28 (35)	7 (21)	0.139
**Microbiology (*n* (%))**						
Gram +	42 (48)	9 (31)	0.116	43 (54)	10 (30)	0.020
Gram −	46 (52)	14 (48)	0.709	40 (51)	13 (39)	0.277
Fungi	17 (19)	5 (17)	0.804	16 (20)	7 (21)	0.909
**Measurements at diagnosis (median (IQR))**						
SOFA score	7 (7)	10 (3)	0.000	9 (3)	10 (3)	0.351
APACHE score	13 (6)	16 (6.5)	0.000	13 (5)	16 (3)	0.006
Total bilirubin (mg/dL)	0.72 (1.56)	0.99 (1.08)	0.324	0.98 (1.67)	1.27 (1.10)	0.662
Glucose (mg/dL)	157 (65)	159 (97)	0.142	169 (76)	193 (145)	0.258
Platelet count (cell/mm^3^)	131,000 (96,250)	100,000 (131,500)	0.415	149,000 (163,250)	123,000 (137,500)	0.565
INR	1.36 (0.37)	1.31 (0.49)	0.989	1.33 (0.33)	1.31 (0.49)	0.325
ScvO_2_ (%)	72.30 (11.9)	66.70 (17.1)	0.007	70.90 (18.00)	67.00 (19.10)	0.334
C-reactive protein (mg/L)	107.80 (208.4)	186.00 (228.4)	0.012	208.60 (213.50)	184.40 (241.60)	0.417
Procalcitonin (ng/mL)	0.99 (9.82)	5.24 (19.49)	0.276	3.72 (23.10)	8.02 (20.46)	0.775
Lactate (mM)	3.11 (1.86)	4.33 (5.50)	0.004	2.89 (2.11)	5.00 (5.00)	0.003
White Blood cells (cells/mm^3^)	13,370 (10,540)	13,560 (10,490)	0.639	15,470 (11,960)	15,350 (10,605)	0.193
Neutrophils (cells/mm^3^)	11,738 (9803)	12,319 (10,623)	0.585	13,614 (11,310)	12,921 (10,420)	0.192

ICU, intensive care units; SOFA, sequential organ failure assessment; APACHE, acute physiology and chronic health evaluation; INR, international normalized ratio; ScvO_2_, central venous oxygen saturation. Quantitative data are expressed as medians with interquartile range (IQR). Qualitative data are presented as percentages and absolute numbers. A *p*-value ≤ 0.05 was considered to indicate significant differences (bold values).

**Table 3:** AUC values for different biomarkers.

Biomarker	Area	Asymptotic 95% Confidence Interval
SOFA score	0.580	0.456–0.705
APACHE score	0.647	0.543–0.751
Procalcitonin	0.589	0.478–0.699
C-reactive protein	0.444	0.323–0.565
White blood cells	0.447	0.332–0.563
Neutrophils	0.446	0.332–0.560

AUC, area under the curve; SOFA, sequential organ failure assessment; APACHE, acute physiology and chronic health evaluation.

## Supplementary Materials

The following are available online at https://www.mdpi.com/2077-0383/9/5/1276/s1, Table S1: Microarray data.
